# The emergence of clonal hematopoiesis as a disease determinant

**DOI:** 10.1172/JCI180063

**Published:** 2024-10-01

**Authors:** Kenneth Walsh

**Affiliations:** Berne Cardiovascular Research Center and Department of Cardiovascular Medicine, University of Virginia School of Medicine, Charlottesville, Virginia, USA.

Somatic mutations accumulate in all tissues with age. Most of these mutations have no observable effect on the phenotype of the cell. However, when a mutation occurs in a “driver” gene that confers a self-renewal, proliferative, or survival advantage to the cell relative to its neighbors, it can lead to a clonal event. As clones expand, the mutant cells can increasingly affect organ function, disease pathogenesis, and biological aging. Although somatic mosaicism is present throughout the body, the hematopoietic system is disproportionately affected by clonal expansions due to its high rate of cellular turnover. In the process of clonal hematopoiesis, hematopoietic stem or progenitor cells acquire somatic mutations that drive clone growth in the absence of overt hematological changes. This series of Review articles in the *Journal of Clinical Investigation* discusses the ramifications of this phenomenon.

The condition of clonal hematopoiesis is predominantly detected in the elderly. This bias is due to the accumulation of mutations with age and, as discussed in this Review Series by Schleicher et al. (1), clone growth can be facilitated by the age-related degradation of the hematopoietic stem or progenitor cell (HSPC) niche. Mutations in HSPCs are passed on to progeny immune cells, and clonal hematopoiesis is typically detected by deep DNA sequence analysis of peripheral blood leukocytes. Clonal hematopoiesis represents a precancerous state, and individuals with clonal hematopoiesis are more likely to develop hematological malignancies, as described in the Review contribution from Dunn et al. (2). However, for the vast majority of these individuals, the clonal hematopoiesis never advances to a cancerous stage. Thus, this condition has been referred to as clonal hematopoiesis of indeterminate potential (CHIP) or age-related clonal hematopoiesis to distinguish it from the more complex clonal events observed in blood cancers. There are many forms of genome instability that underpin clonal hematopoiesis including single nucleotide variants and small insertions and deletions (indels) in genes that are recurrently mutated in hematological malignancies (3). Frequently, these mutations occur in the genes that encode the epigenetic regulators DNMT3A, ASXL1, and TET2, leading to widespread effects. In addition, clonal hematopoiesis can arise from major chromosomal alterations, including amplifications, deletions, rearrangements, and loss of heterozygosity, and these alterations are more difficult to study in experimental systems because of their size and heterogenous nature. Along these lines, chromosome aneuploidy is prevalent in the hematopoietic system, and mosaic loss of the Y chromosome represents the most commonly acquired mutation in males (4).

The first evidence of clonal hematopoiesis was documented decades ago through the analysis of X chromosome inactivation in females. For example, it was reported that healthy females older than 60 years of age displayed skewing of the maternal to paternal ratio of X chromosome inactivation in leukocytes that is indicative of a clonal expansion, whereas younger females did not appear to exhibit this condition (5). An important advance in our understanding of clonal hematopoiesis was made by Busque et al. (6), who showed that older females with X chromosome inactivation skewing were more likely to harbor mutations in *TET2*. These findings were markedly extended by a pair of studies published in 2014 that used whole-exome sequencing of peripheral blood leukocytes to show that aging is associated with the expansion of clones that harbor mutations in any of the multiple driver genes (7, 8). These studies also provided the first evidence that clonal hematopoiesis is associated with an increase in all-cause mortality after adjustment for traditional risk factors. Furthermore, the increased mortality could not be entirely reconciled with the increased risk of hematologic malignancy (8), suggesting that clonal hematopoiesis may be associated with other fatal age-associated diseases. These findings have transitioned the study of clonal hematopoiesis from an underappreciated field to a burgeoning area of research that has provided new insights about the basis of age-associated diseases and possible therapeutic strategies.

Studies have associated clonal hematopoiesis with the incidence and prognosis of a multitude of diseases. Because cardiovascular disease (CVD) is a major cause of morbidity and mortality, it is not surprising that many studies have found associations of clonal hematopoiesis with diseases of the heart and blood vessels. As discussed by Oren et al. in this Review Series (9), clonal hematopoiesis represents a new risk factor for atherosclerotic CVD that appears to be as impactful as the traditional risk factors that have been appreciated for decades. Clonal hematopoiesis has also been linked to chronic kidney disease, infectious disease, acquired metabolic disease, autoimmune conditions, and solid organ tumors, among others (Figure 1) (3). Furthermore, studies have identified an association between clonal hematopoiesis and biological aging as assessed by the measurement of genome methylation “clocks” (10, 11).

In contrast to age-related clonal hematopoiesis, therapy-related clonal hematopoiesis (t-CH) describes the clonal expansions that occur in individuals who have been subjected to genotoxic stressors, typically during cancer therapy. As discussed by Takahashi et al. (12) in this Review Series, t-CH can arise from mutations in genes, including *TP53* and *PPM1D*, that are components of the DNA damage response (DDR) pathway. While cancer therapies can lead to the formation of de novo mutations, recent evidence suggests that genotoxic exposure leads to the amplification of preexisting clones that harbor mutations in genes that encode the DDR pathway proteins. In this scenario, the genotoxic stress creates an environment in which the mutation confers a survival advantage to the HSPC. t-CH can be viewed as being more aggressive in terms of clonal expansion rates and, as such, can be detected in young childhood cancer survivors (13). t-CH can increase the risk for therapy-related myeloid neoplasm, and experimental data suggest that it could contribute to the late-onset cardiotoxicity that is observed in cancer survivors who have been treated with genotoxic agents (14).

Experimental studies have provided ample evidence for causal relationships between clonal hematopoiesis and diseases. These studies have predominantly focused on murine systems in which bone marrow harboring mutations in putative driver genes is transplanted into WT mice, and the recipient mice then undergo interventions that model disease processes (15). These studies have revealed that multiple driver genes can confer a proinflammatory phenotype to leukocytes (3). Thus, clonal hematopoiesis is believed to facilitate disease pathogenesis through the development of a chronic inflammatory state, a paradigm that is consistent with the inflammatory component to many diseases and the observation of “inflammaging” that occurs with biological aging. Experimental studies of the *TET2* driver gene in models of CVD made the first causal connections between clonal hematopoiesis and nonhematological diseases (16, 17). These murine studies found that inactivating mutations in hematopoietic cell *Tet2* lead to higher expression of proinflammatory proteins in myeloid cells, including the IL-1β cytokine. Notably, inhibition of the NLRP3 inflammasome, which produces the mature form of IL-1β, can largely reverse the pathogenesis conferred by hematopoietic *Tet2* mutations in models of CVD and other diseases. Thus, on the basis of these findings, clinical studies are underway to test whether patients who harbor *TET2* and other forms of clonal hematopoiesis will have superior responses to pharmacological agents that inhibit the inflammasome and other inflammatory mediators (9). Although preliminary, promising studies have reported that treatment with an anti–IL-1β antibody preferentially reduces the risk of major adverse cardiovascular events in patients with *TET2*-mediated clonal hematopoiesis (18).

While experimental studies have provided evidence that clonal hematopoiesis can causally contribute to disease progression in many instances, it is worth noting that individuals living with HIV have a greater incidence of clonal hematopoiesis and faster clone expansion (3). These findings indicate that lifelong infections can increase the likelihood of developing clonal hematopoiesis through the creation of an inflammatory state that alters the HSPC environment and favors the expansion of the mutant cell clones. As such, these findings suggest the existence of “reverse causality,” whereby disease conditions can favor the development of clonal hematopoiesis. Elaborating on this, Schleicher et al. (1) review the mechanistic features that contribute to enhanced clonal expansions in the hematopoietic system.

Despite these relatively rapid advances in clonal hematopoiesis research, formidable challenges lie ahead. First, we require a better understanding of what constitutes a clinically relevant clone size. Studies using conventional next-generation sequencing or the analysis of large study populations with minimal phenotypic characterization find that only very large clones, in some cases 10% or greater variant allele fraction (VAF) in peripheral blood leukocytes, are associated with disease pathogenesis. However, advances in ultradeep, error-corrected DNA sequencing have led to the identification of much smaller clones that can be detected at higher frequencies and in younger cohorts (19, 20). These advanced detection methods, combined with the analysis of highly phenotyped cohorts, have revealed that clones of 1% VAF or lower can be associated with disease incidence, burden, and prognosis (21). Furthermore, ultradeep, error-corrected DNA sequencing of leukocytes has led to the realization that many individuals harbor complex combinations of relatively small clones that could potentially synergize to affect overall immune function (22).

Related to these concerns, the field has a limited understanding of how different driver genes can affect disease processes. Many epidemiological studies analyze individuals who are grouped together as being clonal hematopoiesis positive or clonal hematopoiesis negative, and they do not discern the contributions of individual driver genes due to the lack of statistical power. However, it is reasonable to speculate that some driver genes could give rise to clonal events that are highly pathological, whereas other clones would be relatively benign or even protective (9). While available evidence suggests that some of the prevalent driver gene clones appear to be particularly harmful (e.g., *TET2* relative to the other abundant driver gene mutations), current technology is unable to assess the relative pathogenicity of the dozens of driver gene clones that are infrequently observed in populations. Another concern is that many of the clonal events detected in blood cannot be attributed to known clonal hematopoiesis driver genes. This situation was first noted in an early study that used unbiased whole-exome DNA sequence analysis to assess clonal hematopoiesis (7), and subsequent studies suggest that these unidentified clonal events may exceed the frequency of clonal events that can be attributed to the known driver genes (22–24).

Finally, a better understanding of the mechanisms that give rise to clonal events in the hematopoietic system and how these clones differentially contribute to diverse diseases will be required to devise strategies to mitigate the effects of clonal hematopoiesis. Thus, while much additional research is required to translate current findings into clinical practice, clonal hematopoiesis has clearly become a fast-moving area of research with implications for many disease states.

## Figures and Tables

**Figure 1:**
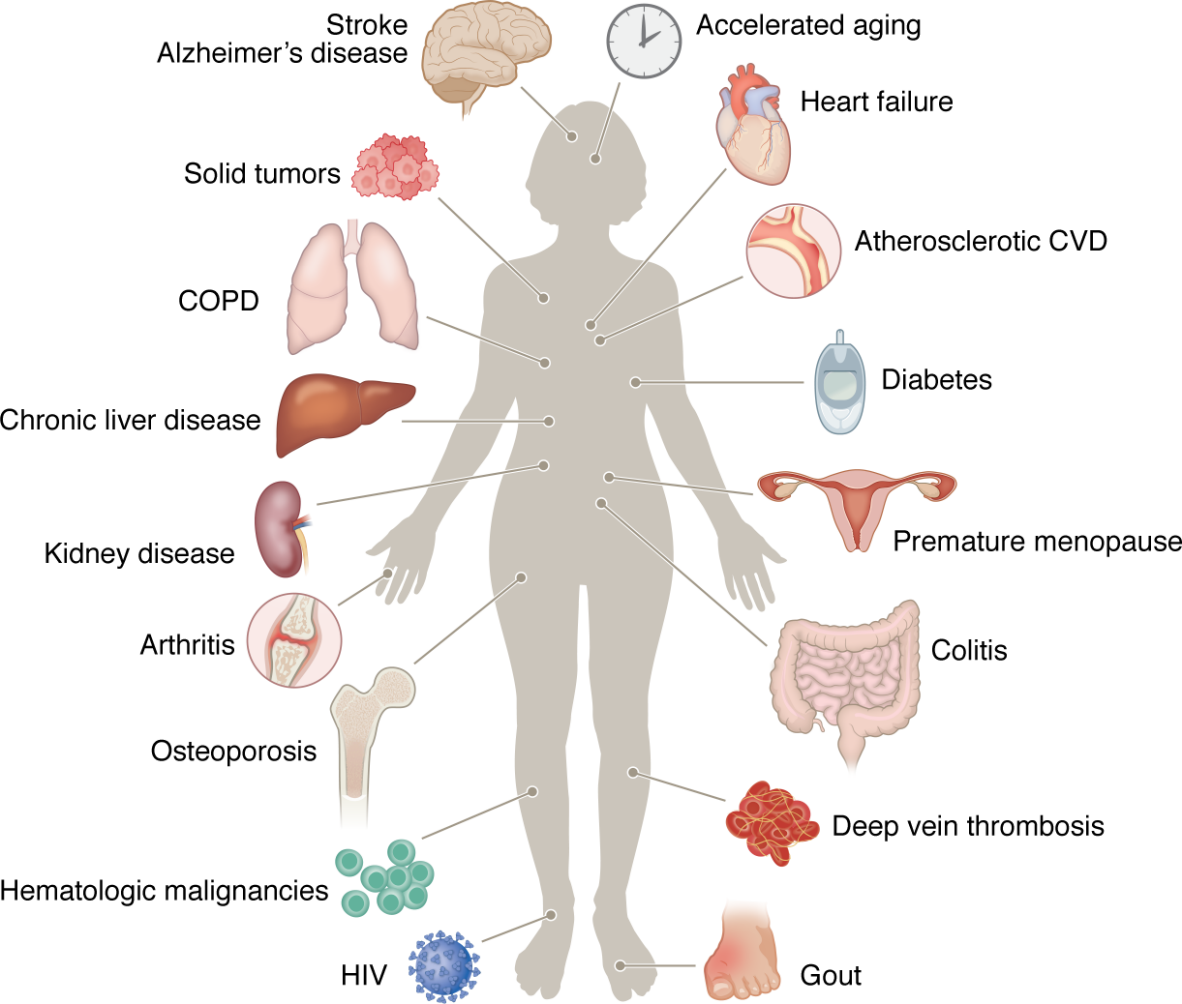
Clonal hematopoiesis is associated with numerous human diseases and conditions. Figure adapted from ref. 3 with permission.

## Version 1 10/01/2024

Electronic publication
